# Predictive Rule for Mortality of Inpatients With Escherichia coli Bacteremia: Chi-Square Automatic Interaction Detector Decision Tree Analysis Model

**DOI:** 10.7759/cureus.46804

**Published:** 2023-10-10

**Authors:** Yudai Nakanishi, Sayato Fukui, Akihiro Inui, Daiki Kobayashi, Mizue Saita, Toshio Naito

**Affiliations:** 1 Department of General Medicine, Juntendo University Faculty of Medicine, Tokyo, JPN; 2 Department of General Internal Medicine, Tokyo Medical University Ibaraki Medical Center, Inashiki, JPN

**Keywords:** escherichia coli, extended-spectrum β-lactamase (esbl), predictive rule, chaid analysis, risk factors

## Abstract

Aim: A predictive rule for risk factors for mortality due to *Escherichia coli *(*E. coli*)bacteremia has not been defined, especially using the chi-square automatic interaction detector (CHAID) decision tree analysis. Here we aimed to create the predictive rule for risk factors for in-hospital mortality due to *E. coli *bacteremia.

Methods: The outcome of this retrospective cross-sectional survey was death in the hospital due to* E. coli* bacteremia. Factors potentially predictive of death in the hospital due to *E. coli* bacteremia were analyzed using the CHAID decision tree analysis.

Results: A total of 420 patients (male:female=196:224; mean±standard deviation [SD] age, 75.81±13.13 years) were included in this study. 56 patients (13.3%) died in the hospital. The CHAID decision tree analysis revealed that patients with total protein level ≤5.10 g/dL (incidence, 46.2%), total protein level ≤5.90 g/dL with disturbance of consciousness (incidence, 39.4%), and total protein level >5.90 g/dL with hemoglobin level ≤11.10 g/dL and lactate dehydrogenase level ≥312.0 IU/L (incidence, 42.3%) were included in the high-risk group.

Conclusions: Appropriate preventative therapy should be facilitated in patients with *E. coli*at a high risk of mortality.

## Introduction

*Escherichia coli* (*E. coli*) is one of the most common types of bacteremia in the community and healthcare settings. According to recent reports, bacteremia caused by *E. coli *has been on the rise worldwide over the past decade and it is a common infection that is encountered in clinical practice [1]. In addition, resistant bacteria, such as extended-spectrum β-lactamase (ESBL)-producing bacteria are also present. Further, the rate of in-hospital bacteremia caused by antibiotic-resistant bacteria is increasing in hospitals in the United States [2].

Previous studies on risk factors for mortality due to* E. coli *bacteremia reported that the most common invasion routes are the urinary and digestive systems [3]. The pulmonary entry route is highly associated with increased mortality due to sepsis [3]. Further, antibiotic resistance did not affect mortality, despite the increasing trend of ESBL-producing bacteria [3].

With the increasing number of infected people and mortality rates, this trend will continue, and healthcare professionals need to classify patients with* E. coli* bacteremia into risk categories but there have been no studies of established, easily understood risk categories. Studies including many risk factors are thought to be useful for physicians who treat *E. coli* bacteria. A similar study was reported on *Staphylococcus aureus* (*S. aureus*) bacteremia from previous preliminary research [4]. This study proposes the predictive model for the mortality of patients with *S. aureus* bacteremia consisting of four predictors. The underlying malignancy, low serum albumin, high glucose, and methicillin resistance were predictors. However, in a similar study of *E. coli*, the report we analyzed in the chi-square automatic interaction detector (CHAID) decision tree analysis was unprecedented.

This study aimed to predict the mortality rate of patients with *E. coli *bacteremia and investigate their involvement in mortality based on their background and clinical factors using the CHAID decision tree analysis.

## Materials and methods

Study design and population

All methods were performed in accordance with the relevant guidelines and regulations. This retrospective study was approved by the Ethics Committee of Juntendo University Nerima Hospital, Tokyo, Japan (approval number: 2020052). Since this study was an observational study, the requirement of written informed consent was waived by the Ethics Commission. Additionally, this study was performed to the Strengthening the Reporting of Observational Studies in Epidemiology guidelines [5]. All patients (only adults, not including children) who were diagnosed with *E. coli* bacteremia from January 1, 2015 to July 31, 2020 were included. The exclusion criteria were *E. coli *bacteremia with polymicrobial pattern. This retrospective, cross-sectional study was performed at Juntendo University Nerima Hospital (490-bed, university-affiliated hospital), Tokyo, Japan. The primary outcome was death in the hospital due to *E. coli* bacteremia. The diagnoses of *E. coli *bacteremia were retrospectively collected from the clinical chart. The diagnosis of bacteremia was based on blood culture results, and the primary site of infection was defined based on not only blood culture but also other culture results and clinical information. The isolation from infection focus is not necessarily a requirement, but at least two physicians were involved in the diagnosis.

Further, when the patients consulted an outpatient, all blood cultures were submitted (all patients were hospitalized within the same day or a few days). If blood cultures were taken repeatedly, only the first culture results for each patient were used for analysis. We identified the bacteria with at least more than two laboratory technicians using a blood cultures autoanalyzer.

The blood culture results were extracted by the chart review and other clinical information was also collected at the same time of submission of blood cultures. The following clinical information was collected: demographic factors (age and sex); primary disease (urinary tract infection, hepatobiliary infection, intraabdominal-pelvic infection, pneumonia that included community-acquired and by aspiration, and abscess); lying condition (body mass index, cancer-bearing, and hemodialysis).

Vital signs (disturbance of consciousness, Glasgow Coma Scale); body temperature; systolic and diastolic blood pressure; heart rate; respiratory rate; and oxygen saturation; intervention (mechanical ventilation); and laboratory data including white blood cell (WBC) counts, hemoglobin (Hb), platelet, and serum parameters (blood urea nitrogen, creatinine, total protein, albumin, total bilirubin, lactate dehydrogenase, aspartate aminotransferase, alanine aminotransferase, sodium, potassium, chloride, glucose, hemoglobin A1c, and C-reactive protein) were also collected.

Statistical analysis

A bivariate comparison of each variable between patients who died in the hospital and survivors was performed using the independent t-test, or the chi-square test. The differences were considered significant (the P-value was below 0.05). The results were then subjected to the CHAID decision tree analysis to identify combinations of the risk factors that were associated with in-hospital mortality. The CHAID decision tree analysis is a data mining technique, and this technique has the salient advantage of advanced graphic presentation for interpretation [6,7]. The CHAID decision tree analysis enables us to effectively deal with whole variables and consecutive partition data. Further, the decision trees use a forward stopping or pruning rule and are the only model used to formulate multiple nodes [7,8]. Unlike other techniques, the significance level can be adjusted for the number of comparisons. The CHAID decision tree analysis has been applied in the medical field [6-9] and has been shown to be superior to the logistic analysis [10]. Furthermore, there is the advantage that complex risks can be evaluated over a single significant index, in another study [11]. For other advantages, results are plain, with little necessary pretreatment, and versatility is high for any data. We can correspond to classification, both recurrences are cited [12]. We thought about all these advantages compositely and decided to choose the CHIAD decision tree analysis as a method of analysis in relation to multiple factors.

In addition, the prediction rules with the CHAID model are visibly intuitive and even more, easy to interpret in clinical settings. The mother nodes and daughter nodes were set to 50 and 25. The multiple 2 × 2 contingency tables between the dependent and independent variables were created first, and then the most significant independent variable in a chi-square test was selected to branch out the decision tree.Thereafter, the categories of each independent variable were merged if they were not significantly different from the dependent variables (cut-off values were established automatically using the chi-square test results) [13]. The goodness-of-fit of the model was examined using the receiver operating characteristic (ROC) curve and its area under the curve (AUC). All analyses were conducted using Statistical Package for the Social Sciences (SPSS) software package version 27.0 (IBM Corp., Armonk, NY).

## Results

As shown in Table 1, 420 patients (male-to-female ratio=196:224; mean±standard deviation [SD] age, 75.81±13.13 years) were included in this study. Fifty-six patients (13.3%) died of sepsis in the hospital. Table 1 shows the characteristics of patients who died in the hospital and survivors and bivariate analysis results. Urinary tract infection (P<0.01), intra-abdominal-pelvic infection (P<0.01), BMI (P=0.05), cancer-bearing (P<0.001), hemodialysis (P<0.01), disturbance of consciousness (P<0.001), axillary body temperature (P<0.01), systolic body temperature (P<0.001), diastolic body temperature (P=0.01), oxygen saturation (P<0.001), hemoglobin (Hb) (P<0.001), blood urea nitrogen (BUN) level (P<0.001), creatinine level (P<0.001), total protein level (P<0.001), albumin level (P<0.001), lactate dehydrogenase (LDH) level (P<0.001), aspartate transaminase (AST) level (P<0.01), alanine transaminase (ALT) level (P=0.03), potassium level (P<0.01), and CRP level (P=0.02) were observed at significantly higher frequencies among those who died in the hospital than the survivors.

**Table 1:** Patient characteristics *P＜0.05; ‡ESBL = extended spectrum β-lactamases; bpm = beats per minute

	Total	Death in hospital	Survivors		P value
Variables	n=420	n=56	n=364	Name of test	
‡ESBL	75 (17.9%)	11 (19.6%)	64 (17.6%)	χ^2^	0.85
Demographic factors					
Age, year, ± SD	75.81±13.13	73.04±10.21	76.24±13.49	t-test	0.09
Female sex, n, %	224 (53.3%)	26 (46.4%)	198 (54.4%)	χ^2^	0.33
Primary disease					
Urinary tract infection, n, %	221 (52.6%)	17 (30.4%)	204 (56.0%)	χ^2^	<0.001*
Hepatobiliary infection, n, %	123 (29.9%)	16 (28.6%)	107 (29.4%)	χ^2^	1.00
Intraabdominal-pelvic infection, n, %	60 (14.3%)	22 (39.3%)	38 (10.4%)	χ^2^	<0.001*
Aspiration pneumonia, n, %	9 (2.1%)	0 (0%)	9 (2.5%)	χ^2^	0.49
Abscess, n, %	10 (2.4%)	2 (3.6%)	8 (2.2%)	χ^2^	0.88
Underlying condition					
Body Mass Index, kg/m^2^, ± SD	21.58±3.92	20.61±3.69	21.72±3.94	t-test	0.05*
Cancer bearing, n, %	139 (33.1%)	32 (57.1%)	107 (29.4%)	χ^2^	<0.001*
Hemodialysis, n, %	21 (5.0%)	8 (14.3%)	13 (3.6%)	χ^2^	<0.01*
Vital signs					
Disturbance of consciousness, n, %	163 (38.8%)	35 (62.5%)	128 (35.7%)	χ^2^	<0.001*
Axillary body temperature, ℃, ± SD	38.19±1.26	37.77±1.23	38.25±1.25	t-test	<0.01*
Systolic blood pressure, mmHg, ± SD	129.67±28.78	114.66±25.98	131.98±28.53	t-test	<0.001*
Diastolic blood pressure, mmHg, ± SD	70.34±16.53	65.14±18.76	71.14±16.04	t-test	0.01*
Heart rate, bpm, ± SD	98.95±20.56	100.11±23.99	98.77±20.01	t-test	0.65
Respiratory rate, n, ± SD	19.27±4.89	19.88±5.34	19.18±4.81	t-test	0.32
Oxygen saturation, %, ± SD	95.29±5.07	92.98±5.76	95.64±4.87	t-test	<0.001*
Intervention					
Mecanical ventilation, n, %	4 (1.0%)	3 (5.4%)	1 (0.3%)	χ^2^	<0.01*
Laboratory data					
White blood cell counts, /μL, ± SD	11,644.29±7,773.03	12,028.57±12,286.06	11,608.24±6,844.45	t-test	0.71
Hemoglobin, g/dL, ± SD	11.59±2.25	9.99±2.00	11.84±2.19	t-test	<0.001*
Platelet, 10^4^/μL, ± SD	13.69±10.38	12.03±125.39	13.95±10.01	t-test	0.19
Blood urea nitrogen, mg/dL, ± SD	29.37±22.41	40.38±28.34	27.68±20.89	t-test	<0.001*
Creatinine, mg/dL, ± SD	1.61±1.79	2.23±1.83	1.51±1.76	t-test	<0.001*
Total protein, g/dL, ± SD	6.39±0.95	5.63±1.03	6.51±0.87	t-test	<0.001*
Albumin, g/dL, ± SD	3.25±0.77	2.65±0.72	3.34±0.73	t-test	<0.001*
Total bilirubin, g/dL, ± SD	1.40±0.32	1.63±1.52	1.36±1.28	t-test	0.16
Lactate dehydrogenase, IU/L, ± SD	411.86±1,181.04	1,055.48±3,162.54	315.58±245.30	t-test	<0.001*
Aspartate aminotransferase, IU/L, ± SD	182.52±830.38	457.45±2,106.98	140.23±331.40	t-test	<0.01*
Alanine aminotransferase, IU/L, ± SD	108.04±406.37	214.84±998.57	91.61±193.65	t-test	0.03*
Sodium, mEq/L, ± SD	136.97±5.94	137.50±10.08	136.89±5.03	t-test	0.47
Potassium, mEq/L, ± SD	3.99±0.67	4.21±1.02	3.95±0.59	t-test	<0.01*
Chloride, mEq/L, ± SD	102.47±5.87	102.02±8.90	102.54±5.27	t-test	0.54
Glucose, mg/dL, ± SD	157.01±100.76	153.13±126.52	157.61±96.44	t-test	0.76
Hemoglobin A1c, %, ± SD	6.24±1.30	6.19±1.53	6.25±1.27	t-test	0.80
C reactive protein, mg/dL, ± SD	11.20±9.83	14.10±9.10	10.75±9.87	t-test	0.02*

**Figure 1:**
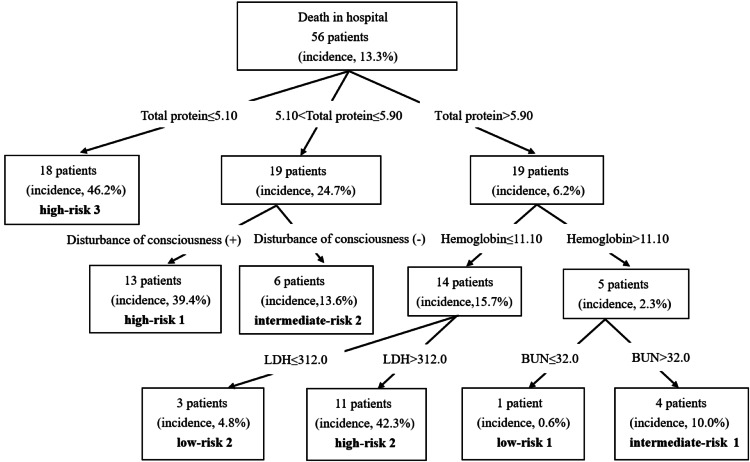
The algorithm for predicting death in patients in hospitals driven using the CHAID decision tree analysis Categories are defined based on bacteremia incidence values as follows: low risk (≤5%), intermediate risk (>5% to ≤20%), and high risk (>20%) LDH = lactate dehydrogenase; BUN = blood urea nitrogen

The algorithm for predicting death in patients in hospitals driven using the CHAID decision tree analysis is shown in Figure 1.

Based on the preliminary research using the CHAID decision tree analysis, patients were categorized into three risk groups; low risk (≤5%), intermediate risk (>5% to ≤20%), and high risk (>20%) [14]. Total protein level, disturbance of consciousness, Hb, LDH, and BUN levels were included in the decision tree analysis, and seven terminal nodes were derived.

**Figure 2:**
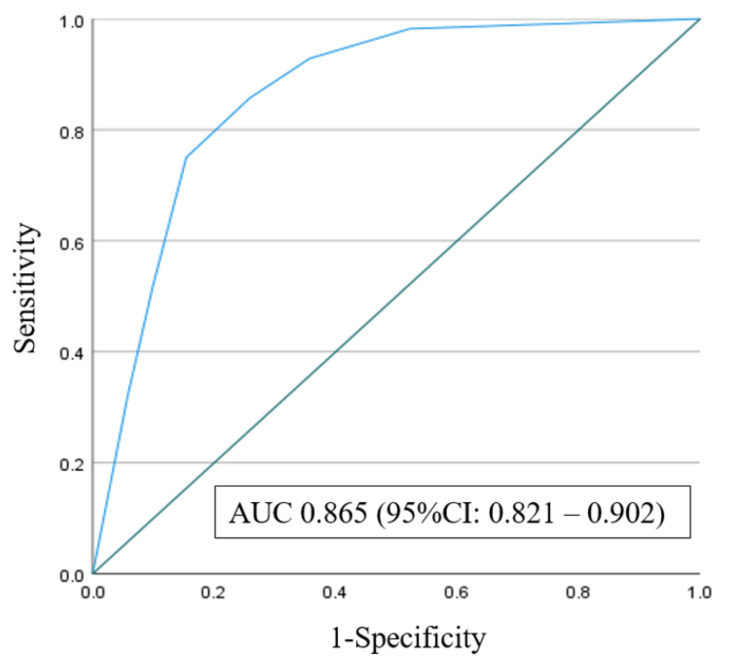
Receiver operating characteristics curve of CHAID-formulated decision tree The AUC was 0.865 (95% confidence interval: 0.821–0.902) CHAID = chi-square automatic interaction detector; AUC = area under the curve

Based on the incidences, the nodes were sorted into low-risk 1(incidence of death in hospital: 0.6%), low-risk 2 (4.8%), intermediate-risk 1 (10.0%), intermediate-risk 2 (13.6%), high-risk 1 (39.4%), high-risk 2 (42.3%), and high-risk 3 (46.2%). Figure 2 shows the ROC curve from the CHAID decision tree analysis. The AUC was 0.865 (95% confidence interval: 0.821-0.902).

Also, the results of the chi-square test for the quick sequential organ failure assessment (qSOFA) score are shown in Table 2. The patients with *E. coli *bacteremia with a qSOFA score of 3 had a significantly higher percentage of in-hospital deaths (P<0.001). The patients with *E. coli* bacteremia with a qSOFA score of 0 had a significantly higher percentage of survivors (P<0.01).

**Table 2:** Results of the chi-square test of the qSOFA score qSOFA = quick sequential organ failure assessment

E. coli bacteremia	qSOFA: score				Total
	0	1	2	3	
Total, n, %	183 (43.6%)	161 (38.3%)	63 (15.0%)	13 (3.1%)	420
Death in hospital	15 (26.8%)	20 (35.7%)	12 (21.4%)	9 (16.1%)	56
Survivors	168 (46.2%)	141 (38.7%)	51 (14.0%)	4 (1.1%)	364
test: χ^2^	<0.01*	0.78	0.21	<0.001*	
*P＜0.05					

**Figure 3:**
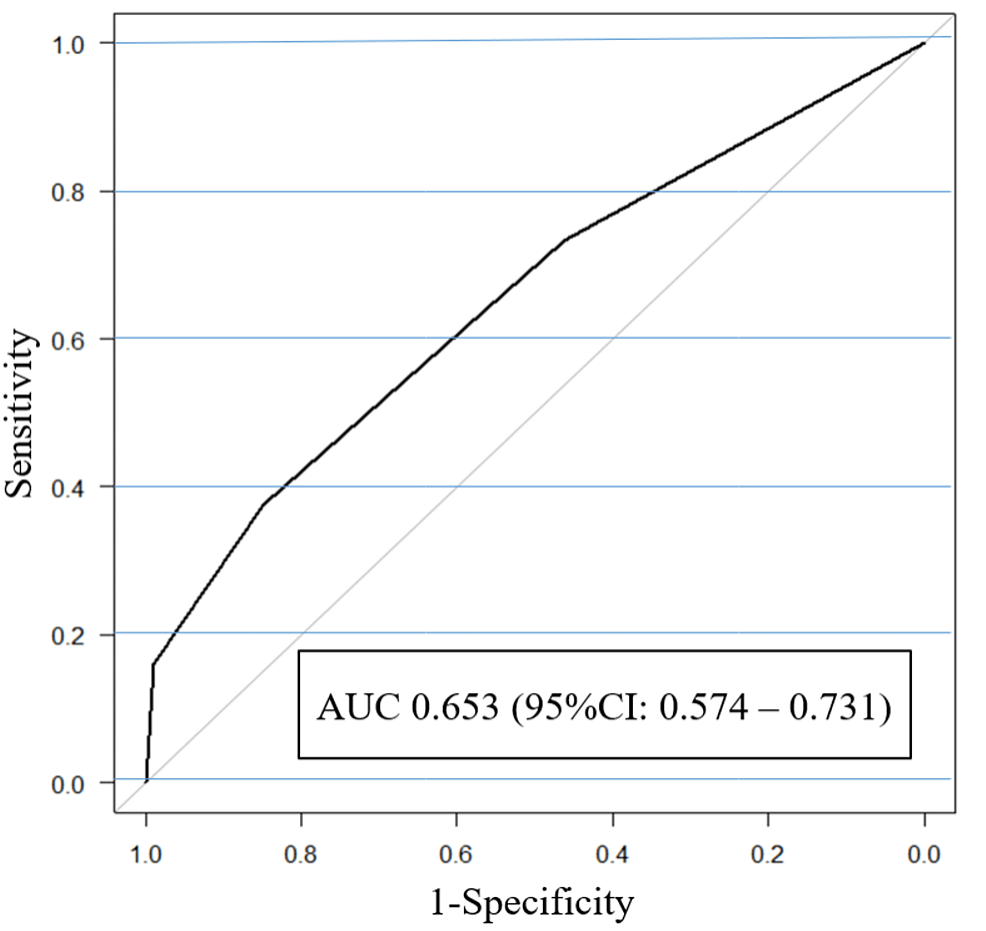
Receiver operating characteristics curve of the qSOFA for the positive risk factors for Escherichia coli bacteremia qSOFA = quick sequential organ failure assessment; AUC: area under the curve

Furthermore, we evaluated the quality of qSOFA using the ROC curve, yielding an AUC of 0.653 and a 95% CI of 0.574-0.731 (Figure 3). A comparison between the AUCs in Figures 2 and 3 indicated that the model obtained using the CHAID decision tree analysis in this study showed greater power than that obtained using the qSOFA [15].

## Discussion

This study is the first report using the CHAID decision tree analysis to predict the mortality of patients with *E. coli* bacteremia. The patients with total protein level ≤5.90 g/dL with disturbance of consciousness (incidence of death in hospital: 39.4%), total protein level >5.90 g/dL with Hb ≤11.10 g/dL and LDH level >312.0 IU/L (incidence of death in hospital: 42.3%), and total protein level ≤5.10 g/dL (incidence of death in hospital: 46.2%), were a high-risk group.

In this study, a total protein ≤5.10 g/dL was identified as a predictor of an intermediate high-risk group for death. In addition, low total protein and albumin levels were significantly associated with mortality and poor prognostic factors for bacteremia [16]. This may be because low nutritional status increases the morbidity of bacteremia, leading to sepsis and subsequent mortality. 

On the other hand, even when total protein levels were within normal, impaired consciousness was associated with an increased risk of death. The concept of sepsis-related encephalopathy has been used to explain the loss of consciousness during sepsis. The pathogenesis is unclear, but possible causes include cerebral ischemia due to circulatory failure, oxidative stress due to biological stress caused by sepsis, and blood-brain barrier disruption due to vascular endothelial cell damage [17]. Several studies have also shown a similar increase in mortality when sepsis is associated with central nervous system symptoms [17,18]. The mortality rate increases with the Glasgow Coma Score, with a score of 15 having a 16% mortality rate, 13-14 having a 20% mortality rate, 9-12 having a 50% mortality rate, and 3-8 having a 63% mortality rate. 

As mentioned above, Hb ≤11.10 g/dL with LDH level >312.0 IU/L was a high-risk group. The mechanisms of Hb reduction in sepsis are varied and may include altered microcirculation, decreased red blood cell (RBC) production, preexisting chronic anemia, hemodilution, and increased RBC destruction due to altered RBC membranes [19]. In addition, the relationship between low initial Hb levels and mortality in conditions such as septic shock has been shown in other studies, and early treatment of patients with low initial Hb levels is thought to contribute to a reduction in mortality [19]. Moreover, LDH is an intracellular enzyme. This is found in almost all organ systems that catalyze the interconversion of pyruvate and lactate and the simultaneous interconversion of nicotinamide adenine dinucleotide (NAD) and NAD-H+ [20]. Therefore, when anaerobic metabolism is increased under conditions of peripheral circulatory insufficiency, an increase in LDH is thought to be the cause. In addition, cytokine-mediated tissue damage increased LDH levels in severe infections [21]. This includes the increased destruction of RBCs. Therefore, elevated LDH may be a finding suggestive of severe infection and may also be a factor in increased mortality.

This study used the CHAID decision tree analysis to examine the factors associated with increased mortality in patients with* E. coli *bacteremia. The qSOFA has been widely used as a rapid diagnostic tool for sepsis, and the qSOFA score was included in the analysis of this study. In qSOFA, if a patient with suspected infection meets at least two of the following clinical criteria: respiratory rate ≥ 22/min, altered mental status, or systolic blood pressure ≤100 mmHg, the patient is significantly more likely to have sepsis and a poor prognosis [22]. Also, this study showed that bacteremia patients with* E. coli *with a qSOFA score of 3 had a significantly higher death rate during hospitalization (P<0.001). Whereas, patients with *E. coli* bacteremia with a qSOFA score of 0 had a significantly higher survival rate (P<0.01). Other studies have reported similar results [23]. However, as shown in the AUC values of the ROC curves shown in Figures 2 and 3, the model based on the CHAID decision tree analysis showed higher detection than the qSOFA of this study. From the above, the CHAID decision tree analysis may be more useful than qSOFA in identifying predictors that may contribute to mortality with *E. coli* bacteremia and referring to results of this study as a prognostic factor for* E. coli* bacteremia patients may contribute to their prognosis.

Furthermore, one of the interesting results of the present study was that ESBL-producing bacteria do not affect mortality in *E. coli* bacteremia. This is similar to what has been reported in several other studies. It is important to note that ESBL-producing bacteria alone do not affect mortality but are significantly associated with delayed initiation of effective antibiotic therapy. The 2007 meta-analysis reported that a delay in the initiation of effective antibiotic therapy is significantly associated with an approximately two-fold increase in mortality from ESBL infection [24]. In the Spanish study, increased mortality among *E. coli *patients in community-acquired infections is not related to whether the bacteria are ESBL-producing but is associated with inappropriate empirical treatment [25]. Currently, the carbapenems are recommended drug for the treatment of ESBL-producing bacteria [26]. In some institutions, the use of second-generation cephem antibiotics such as cefmetazole (CMZ), which are susceptible to ESBL-producing bacteria, can be considered [27]. For ESBL-producing bacteria, our hospital has been using CMZ rather than carbapenem since 2017 due to sensitivity according to the literature [27], and the percentage is 38.6% (29 patients/all 75 patients) of all ESBL-producing bacteria (However, the values up to CRE/CPE, AmpC could not be extracted precisely, this point was the limitation). In this regard, education on antimicrobial therapy is thoroughly provided at our hospital. Blood cultures are submitted when we suspect an infection likely to cause *E. coli* infection, such as urinary tract, hepatobiliary tract, or intra-abdominal infections, and effective antimicrobial agents that can cover ESBL-producing bacteria are promptly administered. This led to early and appropriate treatment, which had no association with mortality from ESBL-producing bacteria. The above shows how important it is to acquire knowledge of appropriate antimicrobial therapies.

This study has several limitations. First, we suspect that the missing values may have affected the CHAID analysis, but the CHAID analysis treats the entire system and the user-missing values for each independent variable as a single category. The given category may or may not subsequently be merged with other independent variable categories for scale and ordinal independent variables, depending on the growing number of criteria [13]. From the above, we believe that the effects of missing values on our results in this study were minimized. This applies to covariate values as well. Furthermore, the present study used a CHAID model, but the risks of bacteremia may also be evaluated by other technique. Second, the patient populations enrolled in this study were limited to a single hospital and a retrospective study. As a next step, we believe that multicenter prospective study with a larger number of patients needs to be conducted. The large sample size may affect the AUC of qSOFA in particular in the current study. While we believe that the treatment of ESBL-producing bacteria was appropriate, we must add that not all patients received the same antimicrobial treatment during the current study period due to the shortcomings of a retrospective study. Third, the study was expected to include other comorbidities such as chronic heart failure and chronic obstructive pulmonary disease, intervention (not only mechanical ventilation but also vasopressor use, abscess drainage such as catheter removal or abscess). Additionally, we could not analyze data on procalcitonin and lactate levels, which may be prognostic factors in bacteremia and sepsis because it was difficult to obtain such data from the chart review. These factors should be analyzed in future studies. Last, we did not calculate the sample size because all patients who were diagnosed of *E. coli* bacteremia from January 1, 2015, to July 31, 2020, were included. For prospective studies in the future, the sample size is to be calculated beforehand.

## Conclusions

This study aimed to predict the mortality of patients with* E. coli* bacteremia and investigate their involvement in mortality rate based on their background and clinical factors using the CHAID decision tree analysis.

Patients with a total protein level ≤5.90 g/dL with disturbance of consciousness (incidence of death in hospital: 39.4%), total protein level >5.90 g/dL with hemoglobin level ≤11.10 g/dL and LDH level >312.0 IU/L (incidence of death in hospital: 42.3%), and total protein level ≤5.10 g/dL (incidence of death in hospital: 46.2%) were included in the high-risk group. Moreover, appropriate preventative therapy should be facilitated in patients with *E. coli* at a high risk of mortality.
